# Editorial: Advances in Therapeutics for Hyperkinetic Movement Disorders

**DOI:** 10.3389/fneur.2022.853084

**Published:** 2022-02-24

**Authors:** Thomas Klopstock, Deborah Hall, Steven Frucht, Emmanuel Flamand-Roze

**Affiliations:** Department of Neurology, New York University Grossman School of Medicine, New York, NY, United States

**Keywords:** hyperkinetic, dystonia, pridopidine, epilepsy, Tourette, NBIA, DBS

## Introduction

This Frontiers in Neurology Research Topic is focused on treatments for hyperkinetic movement disorders and includes papers on diagnosis, review of therapeutics, and original research studies. Hyperkinetic disorders include a diverse group of conditions, with this Research Topic including a broad range of disorders such as Huntington's disease, dyskinetic cerebral palsy in children, and functional dystonia.

## Presenting the Contributing Articles

Kleimaker et al. review current concepts regarding the pathophysiology of Gilles de la Tourette syndrome (GTS) and respective treatment approaches. They explain why non-invasive brain stimulation, in particular repetitive transcranial magnetic stimulation (rTMS) and transcranial direct current stimulation (tDCS), are promising therapeutic options in otherwise treatment-refractory cases, and could be applied intermittently during times of tic exacerbation. While evidence so far is very limited, Kleimaker et al. give recommendations for the design of future studies.

Iankova et al. review the heterogeneous group of currently 15 monogenetic disorders that are embraced under the umbrella term Neurodegeneration with Brain Iron Accumulation (NBIA), and focus on the challenges in treating these ultrarare conditions. While current treatment remains largely symptomatic, several disease-modifying therapies are being developed. Iron chelation is an obvious approach, has shown marked radiological reduction of iron load in the basal ganglia but only a trend to slowing of disease progression in the NBIA subtype pantothenate kinase-associated neurodegeneration (PKAN). More sophisticated treatment approaches, reaching from bypassing the defective enzyme to gene therapy, are currently investigated *in vitro*, in animal models and in clinical studies.

There are two original Research Topics focused on hyperkinetic movement disorders populations investigating tetrabenazine for treatment and battery preference for deep brain stimulator recipients. In the first, Scalise et al. conducted a retrospective longitudinal study in children with dyskinetic cerebral palsy treated with tetrabenazine. The primary purpose of the study was to collect data on efficacy of tetrabenazine using a new standardized rating scale for movement disorders in childhood. The researchers found that a customized regimen of tetrabenazine, especially in children with DSP who were already on treatment, was ideal and this allowed for dosage adjustment when side effects were encountered. This Research Topic also showed that the Movement Disorders—Childhood Rating Scale was suitable to detect suitable changes during this observational study.

In the second Research Topic, Qiu et al. administered a questionnaire using a web-based format to determine satisfaction and preference for rechargeable (r-IR) vs. non-rechargeable (nr-IPG) batteries for deep brain stimulators (DBS) placed for dystonia or Tourette syndrome. The survey was conducted in 100 patients who had DBS placement in Shanghai and the results showed that satisfaction did not differ significantly between patients with r-IPGs or nr-IPGs. In addition, patients with hyperkinetic movement disorders were able to recharge their devices, similar to Parkinson's disease patients with r-IPG. However, patients considered economic factors first when choosing between an r-IPG or nr-IPG as r-IPGs are less affordable in China.

In their beautiful review, Méneret et al. summarize a critical group of unusual hyperkinetic movement disorders not to be missed. Autoimmune syndromes such as antiphospholipid syndrome, NMDA-receptor mediated encephalitis and anti-IgLON5 disease are treatable and even reversible if recognized and managed early. A variety of infectious agents (toxoplasmosis, Whipple's disease, syphilis, and tetanus) can present with hyperkinetic movements disorders, and the astute clinician can make a profound difference in outcome with early recognition. Drug-induced and potentially reversible hyperkinetic movement disorders are common (lithium, antipsychotics, and psychostimulants).

In the next review, Frucht et al. present a comprehensive review of functional dystonia, one of the most common presentations of functional movement disorders. Functional dystonia may affect any part of the body (oromandibular, cervical, arm, leg, and trunk), and each form has characteristic clinical features that aid in diagnosis. Effective management depends on a secure diagnosis, compassionate care team, and comprehensive care plan that involves physical therapy, occupational therapy, counseling, and psychiatric input. This treatable and reversible hyperkinetic disorder demands that the treating clinician do everything possible to give patients the best possible chance at recovery.

Pridopidine is a potential drug candidate for the treatment of Huntington's disease (HD). This Research Topic of Frontiers in Neurology propose a meta-analysis of published randomized controlled trials to provide further insights into the efficacy and safety of pridopidine in HD patients (Chen et al.). A mild improvement of Huntington's Disease Rating Scale total motor score was found only at a dose ≥90 mg/day with an increase of adverse events, such as nasopharyngitis and insomnia. This suggests that additional investigations are needed to determine the potential role of this drug in the therapeutic strategy in HD.

In a comprehensive review, de Gusmão et al. nicely discuss the growing number of gene defects that can account for the phenotypic combination of movement disorders and epilepsy, and how this shed light on the pathogenesis and management of these disorders. They emphasize the need for careful clinical phenotyping for interpretating the results of genetic investigations in this setting.

## Conclusion

This compilation of articles expands our understanding of novel therapeutic interventions being developed for hyperkinetic movement disorders.

## Author Contributions

All authors listed have made a substantial, direct, and intellectual contribution to the work and approved it for publication.

## Conflict of Interest

The authors declare that the research was conducted in the absence of any commercial or financial relationships that could be construed as a potential conflict of interest.

## Publisher's Note

All claims expressed in this article are solely those of the authors and do not necessarily represent those of their affiliated organizations, or those of the publisher, the editors and the reviewers. Any product that may be evaluated in this article, or claim that may be made by its manufacturer, is not guaranteed or endorsed by the publisher.

